# Functional Rotation of the Transporter AcrB: Insights into Drug Extrusion from Simulations

**DOI:** 10.1371/journal.pcbi.1000806

**Published:** 2010-06-10

**Authors:** Robert Schulz, Attilio V. Vargiu, Francesca Collu, Ulrich Kleinekathöfer, Paolo Ruggerone

**Affiliations:** 1 School of Engineering and Science, Jacobs University Bremen, Bremen, Germany; 2 Istituto Officina dei Materiali del CNR, UOS SLACS and Dipartimento di Fisica, Universita' degli Studi di Cagliari, Monserrato, Italy; University of Illinois, United States of America

## Abstract

The tripartite complex AcrAB-TolC is the major efflux system in *Escherichia coli*. It extrudes a wide spectrum of noxious compounds out of the bacterium, including many antibiotics. Its active part, the homotrimeric transporter AcrB, is responsible for the selective binding of substrates and energy transduction. Based on available crystal structures and biochemical data, the transport of substrates by AcrB has been proposed to take place via a functional rotation, in which each monomer assumes a particular conformation. However, there is no molecular-level description of the conformational changes associated with the rotation and their connection to drug extrusion. To obtain insights thereon, we have performed extensive targeted molecular dynamics simulations mimicking the functional rotation of AcrB containing doxorubicin, one of the two substrates that were co-crystallized so far. The simulations, including almost half a million atoms, have been used to test several hypotheses concerning the structure-dynamics-function relationship of this transporter. Our results indicate that, upon induction of conformational changes, the substrate detaches from the binding pocket and approaches the gate to the central funnel. Furthermore, we provide strong evidence for the proposed peristaltic transport involving a zipper-like closure of the binding pocket, responsible for the displacement of the drug. A concerted opening of the channel between the binding pocket and the gate further favors the displacement of the drug. This microscopically well-funded information allows one to identify the role of specific amino acids during the transitions and to shed light on the functioning of AcrB.

## Author Summary

In nature, bacteria have to resist several toxic threats to be able to survive, from bile acids in intestines up to antibiotics. The *Escherichia coli* bacterium, which usually is a commensal inhabitant of human intestines, can also acquire pathogenic properties which would harm the human body. To dispose of toxic compounds, *E. coli* has developed a protein machinery which is called “efflux pump”. Here, we studied the dynamics of the transporter protein AcrB, a component of the *E. coli* major efflux system, in complex with an antibiotic (doxorubicin). We used computer simulations to complement the existing experimental data. Our purpose was to gain more detailed insights into the pumping mechanism at the molecular level. In our simulations the drug leaves the binding pocket upon induction of functional rotation in the protein, although a complete extrusion was never observed. A peristaltic motion, which starts with a zipper-like closure of the interior of the protein, is an important step for the extrusion of the drug. Interestingly, such a peristaltic mechanism of pumping has been suggested before on the basis of structural data. The molecular details obtained in this study shall deepen the understanding of the functioning of the efflux pump.

## Introduction

The acquisition of multidrug resistance (MDR) by bacteria, both in hospitals and in the community, has become one of the most serious impediments to improved healthcare [1]–[6]. Unfortunately, MDR is not restricted to antimicrobials, being common to antimalarials, herbicides, and anticancer agents as well [4]. A key role in MDR is played by efflux pumps, which feature some characteristics with respect to common membrane transport systems [7], [8]. Indeed, while the latter typically are highly specific for their substrates, efflux pumps possess a broad specificity for a wide range of chemically unrelated molecules and drugs [3]–[5], [9], [10].

MDR is of particular concern in Gram-negative bacteria, since this class includes several human pathogens, e.g., *Pseudomonas aeruginosa* and *Enterobacter aerogenes* [4], [5], [11], [12]. Genetic and biochemical data [12]–[17] have shown that the major efflux systems in these bacteria constitute a tripartite complex spanning the periplasmic space across both the inner and the outer membrane [18], [19]. These efflux systems contain an inner-membrane transporter of the Resistance-Nodulation-Division (RND) superfamily [6], [20], [21] and extrude a large variety of toxic compounds, including novel experimental antimicrobials [22]. In *E. coli*, the system is composed of the outer membrane protein TolC [23], the periplasmic membrane fusion protein AcrA [24], [25], and the inner-membrane cation-drug antiporter AcrB [26], [27].

**Figure 1:**
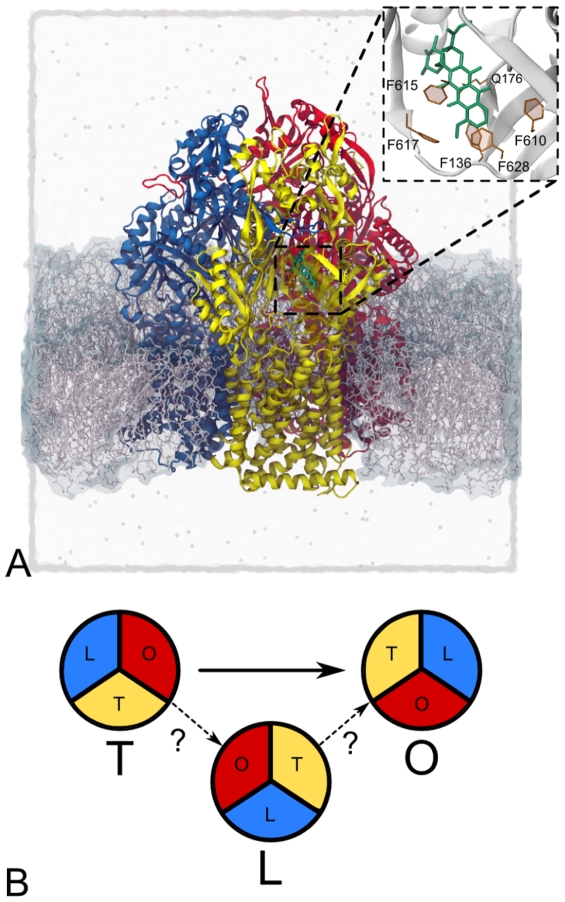
Simulation setup. A) Simulation box: doxorubicin (green) in the binding pocket of AcrB (L, T, and O monomers are shown in yellow, blue and red, respectively) embedded in a POPE lipid bilayer (gray). The system is immersed in a 0.1M KCl solution (ions as spheres, water as transparent box). A magnification of the binding pocket is shown in the upright inset. B) Schematic representation of the functional rotation steps performed here: 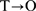, i.e., the one proposed on the basis of the x-ray structures, and 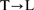.

The active part of the efflux pump - AcrB - (see Fig. 1A) is primarily responsible for the uptake and selectivity of the substrate as well as for the energy transduction. Its structure has first been solved as a symmetric homotrimer [27]. Three main domains have been identified in each monomer: the transmembrane domain embedded in the inner membrane, which provides the energy using the transmembrane proton gradient; the pore/pumping domain in the periplasm, that is supposed to contain the gates from which substrate uptake and extrusion toward TolC occur [28], [29]; and the TolC docking domain, containing a central funnel and presumably being in contact with TolC. More recently, AcrB has been crystallized as an asymmetric trimer with [30] and without [31], [32] a substrate bound in the interior of the protein. Each monomer was found in a different conformation (hereafter Loose, Tight, and Open, or L, T, and O, respectively, following Ref. 31). In the structure of Murakami *et al.* [30], the drugs doxorubicin and minocycline were found inside a binding pocket in the T monomer. The three conformations of AcrB have been interpreted as states of a transport cycle, schematically represented in Fig. 1B, which occurs via a three-step *functional (not physical) rotation* [30], [31]. Following the hypotheses formulated in Refs. 11, 28, 29, the substrate should enter the pore domain of the transporter via the L and/or T monomer, either from open clefts in the periplasm or through grooves between helices at the interface between pore and transmembrane domain. Then, the substrate should accomodate into a binding pocket when the monomer assumes the T conformation and move out toward the TolC docking domain upon a subsequent change to the conformation O. The proposed mechanism is primarily based on the available crystal structures, and has been confirmed only indirectly [33], [34]. Recently, it has been subject of a critical review [28]. In particular, it is not known how conformational changes of the protein cause the extrusion of the drug, and to what extent diffusion is important in the process.

A direct and atomistic-level description of the interplay between structure and dynamics of the conformational changes might render the proposed pictures of the function less speculative and allow to acquire knowledge on the structure-dynamics-function relationship. Such insights will be of support for the analysis of the huge amount of genetic, mutagenetic, and other biochemical data on RND transporters [3]–[6], [9], [11], [35]. Additionally, they will constitute valuable information for a structure-based design of efficient antibiotics and inhibitors, by identifying possible target and key domains in the different steps of the extrusion process. In this respect, molecular dynamics (MD) simulations already pinpointed important atomic-level details of the functioning of TolC [36], [37] and MexA [38], a homologue of AcrA. Despite the importance of AcrB, no computational studies of this transporter have been reported so far.

Here, we present a first attempt to address the relationship between functional rotation and extrusion of substrates: starting from the structural information available on the complex of AcrB with doxorubicin [30], we simulated the proposed final extrusion step of the functional rotation. This was done via targeted molecular dynamics (TMD) simulations [39], which enables to mimic the conformational changes of the protein without explicitly considering the proton transfer and the related energy transduction, which would require the introduction of quantum-mechanical calculations. TMD has been successfully applied to study conformational changes in large systems as 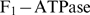 [40] and MurD [41], and it has recently been shown to provide reliable transition paths as compared to other methods used to sample conformations of proteins [42]. Note that in this work we are not investigating the issue of substrate specificity of AcrB, which would require additional compounds to be considered. In the following we show that doxorubicin leaves the binding pocket upon induction of functional rotation, although its total extrusion into the TolC docking domain is not observed. The main aspects as well as the possible limiting factors of the process are discussed. Furthermore, we investigate the presence of a peristaltic-like mechanism and characterize its underlying atomic rearrangements.

## Results

The quite modest success in the fight against bacteria endowed with the MDR machinery [1] is partly due to the lack of knowledge of the connections between structural and dynamical features, which determine the function of the efflux systems at the molecular level. In an effort to shed some light thereon, we investigated to what extent drug motion is related to the suggested extrusion step of the functional rotation. To this end, we started from the T state for the occupied monomer, wherein the anthracycline antibiotic doxorubicin was co-crystallized previously [30]. Then, the transition 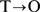, i.e., the final extrusion step of the functional rotation proposed in Refs. 30–32, is enforced via TMD. Note that such a short notation highlights the conformational changes of the T monomer, but all other monomers are also forced toward their corresponding conformations, i.e., 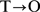 is equivalent to 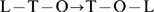 (see Fig. 1B). The main results presented below stem from simulations in which all heavy atoms of the protein were steered. To estimate possible side effects of the TMD approach, we further discuss the results from TMD simulations where only 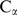 atoms were steered (see Tab. S1 for a list of all the performed TMD simulations). During all the TMD simulations, doxorubicin is not steered by the external bias that drives the conformational changes of the protein, but it is free to move. In the T conformation, the suggested entrance from the periplasm to the binding pocket is opened and the exit gate toward the central funnel is closed (see Fig. 1B). In the O conformation, the closing/opening configuration is toggled, hence the substrate should be able to move out of AcrB toward TolC [32].

### Displacement of the Drug

Initially, doxorubicin is found within a binding pocket which is located between 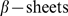 of the subdomains PC1 and PN2 [30] and formed by the residues F136, Q176, F610, F615, F617, and F628 [35], as shown in the inset of Fig. 1A. During the transition, these subdomains undergo conformational changes, thereby displacing the drug. In general, the whole binding region, which contains the described binding pocket has a quite large internal volume, probably with more than one binding site [4].

**Figure 2:**
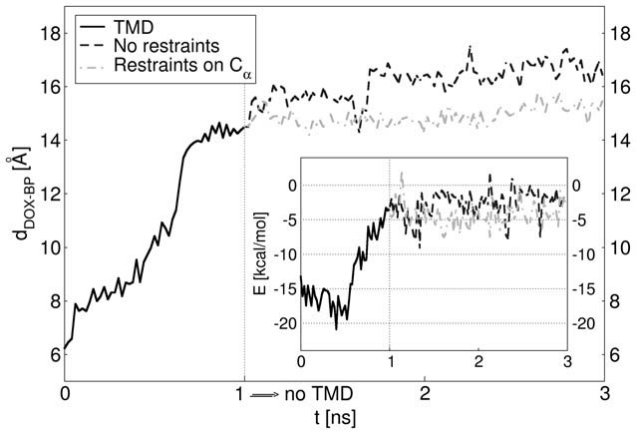
Movement of doxorubicin within the protein. Plot of the distance between the CoMs of doxorubicin and the binding pocket, 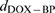 (solid curve), as a function of the simulation time during the 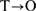 transition. The curve represents an average over simulations with 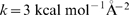 and different initial velocities (see Methods). Also shown are the same distances during ensuing standard MD simulations with restraints on 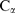 atoms of the protein (dot-dashed), and without restraints (dashed). The interaction energy is given in the inset.

Fig. 2 displays the calculated distance between the centers of mass (CoMs) of the binding pocket and doxorubicin, 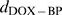, as a function of the simulation time, along with the values of the interaction energy. At the end of the TMD simulations, the RMSD of the protein with respect to the target (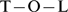) is 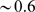 Å (Fig. S1), indicating that the transition has been accomplished. Furthermore, the substrate has moved away from the binding pocket by a total distance of 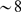 Å toward the gate to the central funnel, formed by the residues Q124, Q125, and Y758 [32]. As shown in the inset of Fig. 2, the interaction energy increases significantly as the transition proceeds, thereby denoting an unbinding event.

**Figure 3:**
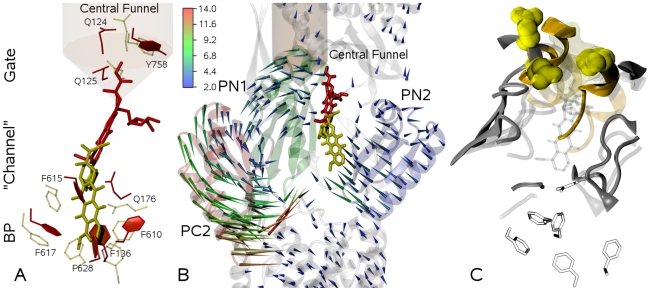
Drug displacement and conformational changes of the protein along the transition 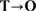. A) Movement of the drug from the binding pocket toward the gate (yellow and red represent the initial and final configuration, respectively). It can be seen from the comparison of the two conformations that residues F136 and F628 in the bottom of the binding pocket and F615 and Q176 in the upper part, are mostly involved in the squeezing of the binding pocket; interestingly residue F610, which mutagenesis experiments have shown to be important for the activity of the pump, seems not directly involved in pumping the substrate out of the binding pocket; B) Porcupine plot of the conformational changes of the subdomains in the pore domain (arrows represent the displacement, in Å, from the initial position of the 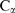 atoms of each amino acid, colored according to the color scale bar). Important subdomains are highlighted: PN2 (transparent blue), PN1 (transparent green), and PC2 (transparent red). As PC1 lacks major changes, it is omitted for clarity. The movement of the drug toward the central funnel is also shown (color code as in panel A); C) Opening of the gate (residues Q124, Q125, and Y758, yellow space-filling representation) and of the BP-Gate path (backbone representation, formed by residues 48–50, 85–89, 126, 163, 177–181, 273–276, and 767–772 of the occupied monomer T - gray - as well as residues 67–70 and 113–117 of the neighboring monomer O - orange, see also text). Transparent and solid representations refer to initial and final states of the 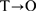 step, respectively. This allows to better appreciate the opening of a path towards the TolC docking domain. For the sake of clarity when compared to the other panels, the final conformations of doxorubicin (transparent stick representation) and the binding pocket (black-and-white sticks) are also shown.

The initial and final positions of the drug of one of the TMD simulations are shown in Fig. 3, as well as the structural changes of the binding pocket and of the gate to the central funnel. The displacement of doxorubicin toward the gate is confirmed by the profile of the distance between the CoMs of the three residues forming the gate and that of doxorubicin (data not shown). During the 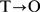 transition, this distance decreases by 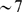 Å, which is a clear indication of the movement of the drug along the path that was identified by Sennhauser *et al.* [32]. Note that the magnitude of displacement is essentially independent of the initial orientation of doxorubicin within the binding pocket (see Fig. S2). Additionally, the obtained displacements are almost insensitive to randomly reinitializing the initial velocities of all atoms or to extending the simulation time to 5 and 10 ns (Fig. S3). In these longer simulations, the major movement of doxorubicin occurs at a different relative time, with respect to the total TMD simulation time, although the final displacements are very similar to those seen in the shorter runs. This indicates a minor dependence of our general results on the simulation time and fortifies the reliability of our calculations.

To assess the stability of doxorubicin in the final position at the end of the TMD runs, we performed two sets of four standard MD simulations starting from the final TMD configurations (see Figs. 2 and S4). In the first case, we restrained the 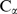 atoms of the protein, in order to keep the backbone in the final 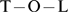 conformation. In the second one, we left the system completely unrestrained and free to relax. In half of the unrestrained simulations, the drug moves further away from the binding pocket by 2 Å in the direction of the gate; in all the remaining runs (2 unrestrained and 4 restrained), it oscillates around its final TMD position. Nevertheless, doxorubicin does not move back toward the binding pocket in any of these simulations.

From the visual inspection of the final position of doxorubicin as shown in Figs. 3A and B, it is clear that the drug did not enter the central funnel of the TolC docking domain during the 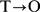 transition. Certainly, the real time scale of the process is out of reach by the computational tools used in the present work, and such a limitation might be a reason for the absence of the complete extrusion in our simulations. Indeed, diffusion could play a relevant role in driving out the drug from AcrB, but this process would occur on a time scale hardly approachable by our protocol. Apart from methodological issues, additional factors have been suggested to be necessary for the full extrusion of the substrate. For example, the necessity of cooperativity effects associated with the binding of a second substrate (absent in our simulations) to a neighboring monomer has been invoked to interpret kinetic data [43]. Furthermore, a more involved pattern of configurations assumed by the monomers and connected to the extrusion process has also been inferred from the analysis of crystallographic structures [28], [44]. A further possible reason might be the absence of the membrane fusion protein AcrA. Its contribution to the functionality of the efflux system seems to go beyond a *simple* structural linker between TolC and AcrB. Surely, a deeper understanding of this interplay will benefit from the simulations of the entire system exploiting the model recently proposed by Symmons *et al.* [25].

Note that upon induction of the conformational transition 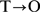, the subdomain PC2 moves inward to close the entrance and is followed by PN1 which opens the exit [31] (Fig. 3B). The distance between the CoMs of PN2 and PC1 declines (see Fig. S5) accompanied by a rotation of the two subdomains, thereby resulting in a shrinkage of the binding pocket. Thus, the motions of the subdomains appear to be the first requirement for the squeezing of the drug out of the binding pocket. However, the largest displacements between the CoMs of the subdomains do occur in the first half of the TMD simulation, while most of the drug displacement is seen in the second half (see Figs. 2, S5, and Video S1). Interestingly, the RMSD of the residues of the binding pocket from the target does not drop much until almost half of the TMD simulation is over. Then, it starts to decrease in correlation with the movement of the drug (Fig. S1), indicating that more specific and local conformational changes are involved in the unbinding of doxorubicin. Thus, our attention focused on the action of specific groups of residues.

### Evidence for Peristaltic Motion

**Figure 4:**
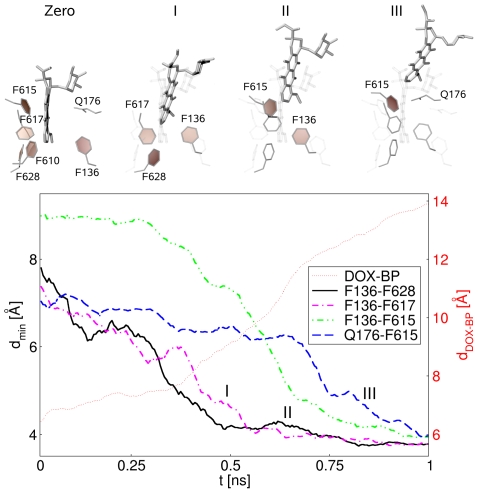
Sequence of the peristaltic squeezing. Upper panel: Snapshots extracted from the MD simulations represent configurations just after the squeezing of certain residue pairs in comparison to the initial state (snapshot labeled “Zero” on the left, solid stick representation). For each snapshot only those residues which are mainly involved in the squeezing step are highlighted. Lower panel: Minimum distances between selected pairs of residues of the binding pocket, 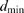, as a function of the simulation time. To better identify different behaviors, we reported running averages (step length equal to 10) of the raw data. The tiny-dotted line represents the distance between the CoMs of the binding pocket and doxorubicin (as plotted in Fig. 2).

A peristaltic pumping was proposed as the extrusion mechanism by Pos and coworkers in 2006 [31]. To identify possible fingerprints of the peristaltic action and correlations between motions of residues and drug displacement, we compared the latter with the evolution of the minimum distances, 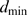, between selected couples of residues in the binding pocket. In particular, we selected those pairs of residues whose distances decline predominantly during the 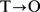 transition, namely F136–F615, F136–F617, F136–F628, and Q176–F615. In Fig. 4, the evolution of their average minimum distances over 5 TMD simulations (lower panel) is shown together with three representative configurations associated therewith (upper panel). Interestingly, the changes in the distances among the selected residues occur in a *step-wise* fashion, with residue pairs at the “bottom” of the binding pocket closing first, and those at the “top” last, producing a zipper-like motion.

The first reduction of 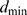 affects the pair F136–F628, but the substrate essentially keeps its position in the binding pocket. Successively, the residues F136 and F617 approach each other, and 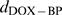 starts to increase (I in lower panel of Fig. 4). The configurations assumed by the three residues are displayed in snapshot I of the upper panel in the same figure. At about one third of the TMD simulation time, the distance of F136–F615 starts to decrease, and this reduction correlates with a large movement of the drug (II in Fig. 4). Note that this is the largest reduction (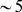 Å) in the distance among the observed pairs of residues. At approximately the same time, the squeezing motion between Q176 and F615 takes place, which is also related to a substantial displacement of the drug (III in Fig. 4). These two amino acids happen to act as a clamp for the planar rings of doxorubicin. The phenyl ring of F615 is atop of one of the rings belonging to the drug and the Q176 amide is on the other side. While the drug is moving toward the gate, the connection between the ring of F615 and the drug is changing from one planar ring of the drug to the next one in a stepwise fashion. Once the residues F615 and Q176 squeeze the substrate out of the binding pocket and thereby close the return path of doxorubicin (see snapshot III in the upper panel of Fig. 4), the drug is able to move further away from the binding pocket.

Concerning each individual TMD simulation, the connection between the zipper-like closure of the binding pocket and the drug displacement can be seen in 4 out of 5 different 1-ns-long simulations (Figs. S6A, C, D, and E). A three-step mechanism can be roughly recognized in the graphs, with a sequential closure of the pairs from the innermost (F136–F628) to the outermost one (Q176–F615). The remaining run of this set (Fig. S6B) could be viewed as a borderline case in which the last step is very short. Despite this, the closure of the binding pocket maintains a basically sequential character, where the outermost pairs (Q176–F615 and F136–F615) close after the innermost ones (F136–F617 and F136–F628). Additionally, three longer TMD simulations (two of 5 ns and one of 10 ns) were performed to assess the dependence of our results from the simulation time (Figs. S6F, G, and H, respectively).

As expected, the molecular details of the process (final displacement, side chain conformation and dynamics) are slightly sensitive to the simulation protocol (see also Fig. S3). Nevertheless, a sequential closure of the binding pocket is still detectable in all panels of Fig S6. In addition to the four out of the five 1-ns-long simulations mentioned above, in one out of the two 5-ns-long ones evidences of a three-step mechanism are recognizable (see caption of Fig. S6 for an extended discussion). Unfortunately, a meaningful statistics, needed for a thorough discussion of the possible limits inherently present in the TMD protocol, is out of range for these longer trajectories.

It is worthwhile to point out that the results of recent mutagenesis experiments [35] have evidenced a significant impact of the mutation F610A on the minimum inhibitory concentration of doxorubicin, while other mutations, including those of the phenylalanines 136, 178, 615, 617, and 628 to alanine, showed smaller effects. According to our simulations, F610 is not prominently involved in the zipper-like action, but might act as binding partner when doxorubicin enters the pore domain, and/or might close the escape from the binding pocket toward the periplasm. Upon mutation to alanine, these actions might not be efficient anymore. Additional studies are required to gain insight into the effect of the F610A mutation.

### Further Essentials of Drug Displacement

To enhance the understanding of the results presented above, we investigated the dynamical coupling between squeezing motions of the binding pocket and other specific residues located beyond it. In particular, we chose those residues lining the path from the binding pocket toward the exit gate. This path (hereafter called BP-Gate path and sketched in Fig. 3C) is formed, with reference to the initial conformation, by the residues 48–50, 85–89, 126, 163, 177–181, 273–276, and 767–772 of the occupied monomer T as well as residues 67–70 and 113–117 of the neighboring monomer O. A series of TMD simulations have been performed in which we kept the BP-Gate path of the T monomer unsteered and forced only the rest of the protein, thereby applying the same bias as in the previous TMD simulations. According to our results, doxorubicin leaves the binding pocket also in these simulations, but the overall displacement is smaller by 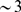 Å with respect to the one shown in Fig. 2. Indeed, the BP-Gate path remains too narrow for doxorubicin to leave the binding region and to move toward the exit gate. Furthermore, the drug is tilted by 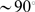 with respect to the final position in Fig. 3A (see Fig. S7), which also hinders further motion toward the gate. This result emphasizes the importance of a concerted opening of the BP-Gate path in addition to the zipper-like closure of the binding pocket.

**Figure 5:**
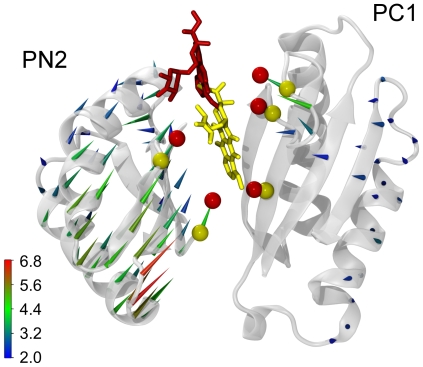
The peristaltic motion of the subdomains. Porcupine plot (as in Fig. 3) of the backbone motion of the subdomains PC1 and PN2 including the 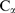 atoms of the residues used in Fig. 4 (initial and final position of these atoms and doxorubicin in yellow and red, respectively).

Since the position and the orientation of amino acids seem to be important for the displacement of the drug from the binding pocket, we further extended our set of simulations to shed more light on this aspect. In the dynamics described so far, all non-hydrogen atoms have been targeted, which corresponds to a forced movement of the side chains during the TMD simulation. To analyze the importance of these movements for the drug displacement in comparison to the influence of the backbone/subdomain, we performed a series of TMD simulations where only the 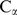 atoms were targeted. This also allowed to test the influence of the biasing force on our results, as a large fraction of the protein is now free to move. We observed a significant displacement of the drug during this set of TMD simulations (see Fig. S8), in qualitative agreement with those obtained by targeting all heavy atoms, hinting at the importance of subdomain motions for the displacement of the substrate (see Fig. 5). On average, the displacement is reduced by 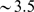 Å with respect to the one reported in Fig. 2. Clearly, the number of possible paths explored by the drug is expected to increase when targeting only the 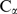, due to the larger flexibility of the protein. Consistently, a displacement comparable to that shown in Fig. 2 is observed only in 3 out of 10 TMD simulations (data not shown). In addition, in some of the 7 remaining runs, doxorubicin does not move straight toward the exit gate, but also turns slightly aside where the interior along the BP-Gate path leaves space to roam. These results indicate that the arrangement of the side chains is able to significantly influence the maximal displacement of the drug.

Additionally, we performed a simulation with a lower force constant (see Tab. S1). The aim was to obtain an indication of the minimal force required to accomplish the conformational changes in the protein, especially along the BP-Gate path. It turns out that, during the entire TMD simulation, the distance of doxorubicin from the binding pocket is lower by a couple of Å with respect to that in previous simulations with larger force constants (Fig. S9). This is related to a larger RMSD of the binding pocket from the target along the simulation (inset in Fig. S9), which, although very small, has an important effect on the displacement of the drug. In combination with the results of the TMD simulations where only 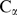 atoms have been targeted, these findings highlight the importance of individual residues including their side-chain conformations for the displacement and subsequent extrusion of doxorubicin.

### Reversing the Direction of the Cycle

**Figure 6:**
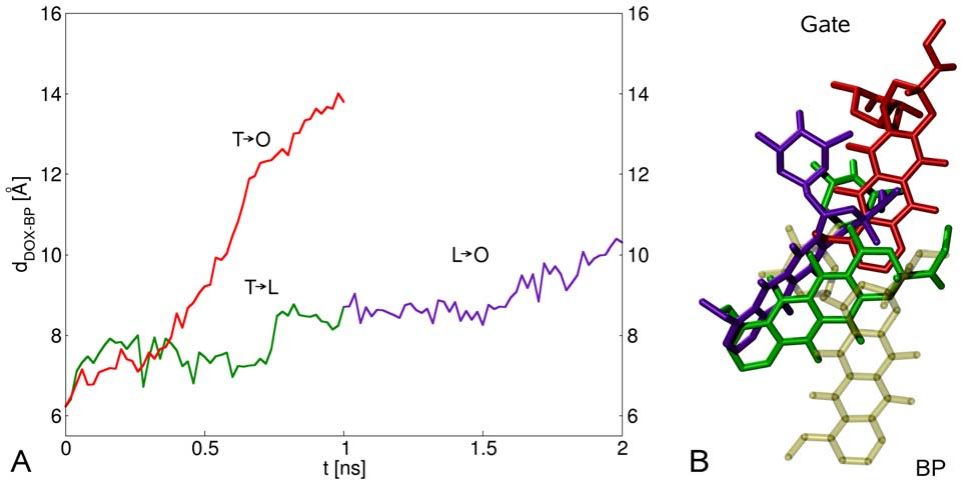
Comparing the two possible directions of the functional cycle. A) Plot of 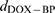 as a function of the TMD simulation time during 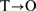 (red curve) and 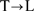 (green curve). The violet curve represents 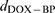 during the step 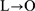 that is the subsequent transition in the reverse cycle (see main text for more details). The values of the force constant for both simulations is 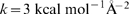. B) Representation of the initial (yellow) and final [color code as in panel A] configurations of the drug.

Analyzing the asymmetric crystal structures of AcrB [30]–[32], it is reasonable to suppose that drugs exit the transporter from the monomer in the O conformation. Therefore, we have considered the direct transition 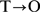 at first. However, the possibility of a functional transition from T to O via the L conformation cannot be ruled out *a priori*. Thus, we carried out simulations for the two steps of the reverse cycle direction, i.e., 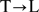 and 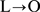 (Fig. 6). The investigation is important for two reasons. Firstly, the direction 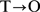 has been suggested to be the functional one from analyses of structural data [30]–[32], but it lacks a direct proof. Secondly, the comparison between the two directions should give a better picture of the conformational changes and drug-amino-acid interactions which are mainly involved in the displacement of the drug.

Interestingly, the specific movements of PN2 and PC1, which have been described above as responsible for the shrinkage of the binding pocket during 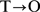, can also be observed during 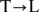 (data not shown). Indeed, the substrate tends to leave the binding pocket in both cycle directions. However, in contrast to 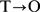, the drug displacement never exceeds 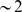 Å for 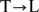, hence doxorubicin does not approach the exit gate. This can be attributed to the quite large internal volume of the binding region [4]. Therefore, substrates may exploit their flexibility and change their orientation. Importantly, the drug does not move remarkably during the second step 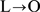 of the reverse cycle as well. Again, this points to the need of a concerted closure of the binding pocket and widening of the channel toward the exit gate.

## Discussion

The molecular dynamics underlying the functioning of many active transporters, which include efflux transporters of the RND family, are not fully understood yet. Although the increasing number of crystal structures permits us to have a closer look at the atomic details of the structure, the dynamical aspects are not caught, and only hypotheses can be advanced concerning the functional mechanisms. MD simulations with atomistic detail are an appropriate tool to investigate structure-function-dynamics relationship in these systems. In this work, we performed TMD simulations to investigate the relations between supposed functional motions in AcrB [30]–[32] and the extrusion of the antibiotic doxorubicin without explicitly considering the energy supply associated with the proton gradient across the inner membrane.

Our results show a detachment of the drug from its initial binding pocket within the T monomer. Moreover, during the 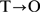 step of the functional rotation, doxorubicin travels by 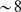 Å and approaches the gate to the central funnel. Importantly, this movement is believed to be part of the suggested extrusion process in AcrB. Our data also support the proposed peristaltic pumping mechanism, and highlight the atomistic dynamics at its basis. In particular, there is evidence to suggest a zipper-like squeezing of the binding pocket, which leads to an unbinding of the substrate along the 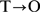 direction of the cycle. The closing of the binding pocket is initially caused by the movements of adjacent subdomains, whereas the rearrangements of individual residues lining the binding pocket strongly influence the detachment of doxorubicin in the end. The molecular details of the extrusion process depend slightly on the TMD simulation protocol (simulation length, targeted atoms), but the main features are robust against these changes.

While investigating the feasibility of the cycle in the reverse direction 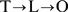, additional simulations have shown a similar squeezing of the binding pocket during the 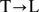 transition. However, no substantial movement of the drug toward the gate has been seen. This could mainly be due to the lack of concerted widening of the BP-Gate path and the exit gate. Moreover, even if such movements do occur during the subsequent 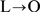 step, they are not coupled to squeezing of the binding pocket, and do not cause any significant movement of the substrate. Altogether, these results strongly points at 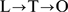 as the legit direction of the functional rotation.

Although a substantial movement of the substrate was seen in our TMD simulations of the 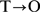 transition, the drug never reached the central funnel of the TolC docking domain, which is a necessary step to achieve the full extrusion of the drug out of AcrB. One possibility to explain this is that further movement of the drug might just be directed diffusion within a confined geometry occurring on a time scale much larger than that captured in the simulations. In addition, the motion of the drug might further be enhanced by attractive interactions between the substrate and residues around the gate or even TolC, or by the presence of other substrates. Finally, the influence of the neighboring monomers as well as the other proteins constituting the efflux pump have to be understood. In the long run, it would be very important to model the whole tripartite efflux pump, i.e., AcrB together with TolC and AcrA. This could complete the picture of the protein-protein interactions involved and their cooperative effects on the drug extrusion. Nonetheless, using the present results it should be possible to obtain a better understanding of the structure-function relationship in RND transporters and its connection to dynamical aspects. Finally, molecular insights on the efflux mechanism in AcrB might be of help for the research on human RND transporter, e.g., the Niemann-Pick C1 disease protein and the hedgehog receptor Patched [28].

## Methods

### System Setup

For our simulation setup, the crystal structure from Ref. 32 was chosen. After addition of hydrogen atoms, a restrained structural optimization was performed. The structure of doxorubicin was taken from Ref. 30 and placed into the system in the same relative position within the binding pocket as original. The latter structure was not used since several loop residues (499 to 512) of the pore domain were not resolved, and the resolution was lower with respect to that in Ref. 32. The combination of a crystal structure with a substrate from another structure was possible since the binding pocket of the protein accommodates the drug very well; indeed, doxorubicin keeps its position during the equilibration. Moreover, the RMSD between the 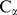 of the structures from Refs. 30, 32 is less than 1 Å. After the placement of the drug, a second relaxation was performed. The protein-substrate complex was inserted into a pre-equilibrated POPE lipid bilayer, which is parallel to the x-y plane, and solvated in TIP3P water with a physiological KCl concentration of 0.1 M. At the end of the buildup phase, all lipid and water atoms which overlapped with the protein were artificially removed; the total number of atoms of the system is 451,962. This setup leads to a periodic box size of 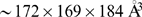.

### Force Fields Parameters

The AMBER force field parm99 [45] was used for the protein, the TIP3P parameters for water [46], and Aaqvist's parameters for the ions [47]. For doxorubicin several parameters were taken from the GAFF force field [48] while the missing ones were generated using modules of the AMBER package [49]. In particular, atomic restrained electrostatic potential (RESP) charges were derived using antechamber, after a structural optimization performed with Gaussian03 [50]. The GAFF parameters for the POPE lipids were generated following the protocol in Ref. [51].

### Simulation Protocol

The unbiased and the targeted MD simulations were both performed with the program NAMD 2.7b1 [52]. After an initial energy minimization, the system was gradually heated up to 600 K and finally quenched to 310 K. All these simulations were performed in the presence of restraints on the phospholipids and the heavy atoms of the the protein. A time step of 1 fs was used for the integration of equations of motion. Furthermore, periodic boundary conditions were employed, and electrostatic interactions were treated using the particle-mesh Ewald (PME) method, with a real space cutoff of 12 Å and a grid spacing of 1 Å per grid point in each dimension. The van der Waals energies were calculated using a smooth cutoff (switching radius 10 Å, cutoff radius 12 Å). Furthermore, the simulations were performed in the NpT ensemble and the temperature was kept at 310 K by applying Langevin forces to all heavy atoms with the Langevin damping constant set to 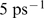. The pressure was kept at 1.013 bar using the Nosé-Hoover Langevin piston pressure control.

The functional rotation was simulated by means of TMD [39] (built-in module of NAMD) which allows to induce conformation changes between two known states. To prevent any hindrance on the T monomer by the neighboring ones, we also steered those toward their next state. Note that the TMD algorithm has recently been demonstrated to produce reliable transition paths as compared to other methods [42]. In this respect, to assess the influence of the biasing force on the dynamics of the system, we performed a series of TMD simulations using different values for the force constant per atom (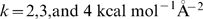) and the simulation time (1, 5, and 10 ns). The results discussed in the main paper refer to simulations of 1 ns with 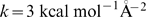, which are consistent with the literature [40], [41], [53]. All TMD simulations performed are detailed in Tab. S1 together with the comparison among distances between CoMs of doxorubicin and the binding pocket and the final positions of the drug (Fig. S2). The setup, the analyses as well as the atomic-level figures, were performed using VMD [54].

## Supporting Information

Figure S1 Drug displacement vs. protein conformational change. Plot of the distance between the CoMs of doxorubicin and that of the binding pocket, d_DOX-BP_, (red full line) and of the RMSD of the whole protein with respect to the target structure (black dashed line), as a function of the TMD simulation time during the T→O transition. The time evolution of the RMSD of the binding pocket from the target structure is also shown (blue dot-dashed line).
(0.68 MB TIF)

Figure S2 Profiles of d_DOX-BP_ for two different conformation and orientation of the drug along the T→O transition. A) Profiles of d_DOX-BP_ for the TMD simulation discussed in the main text (red full line) and for one simulation in which doxorubicin is in a different conformation and orientation within the binding pocket (green dashed line). In the inset is reported the behavior of the interaction energy between the drug and the residues of the binding pocket. Also shown are the two different initial positions - B) top view; D) side view - as well as (D) the final positions of the drug.
(0.40 MB TIF)

Figure S3 Drug displacement vs. simulation parameters along the T→O transition. A) Distance d_DOX-BP_ as a function of the percentage of TMD simulation time for the set of simulations where all heavy atoms have been targeted. To better identify different behaviours, we report running averages of lenght 10 of the raw data. Varying the initial velocities within the set of simulations of same lenght and k value does not remarkably alter the profile of d_DOX-BP_. Extending the simulation time does not sensitively affect the final position of the drug, although the profile of d_DOX-BP_ show some differences with respect to the former set; B) Final positions of doxorubicin in the same set of simulations. The CoMs of the drug are shown as filled spheres to highlight the similar displacements of the drug despite the difference which can be seen in the orientation.
(0.35 MB TIF)

Figure S4 Profile of d_DOX-BP_ after the T→O step. The panel reports the behavior of d_DOX-BP_ in eight post-TMD simulations. Two sets of such simulations (each 2 ns long) have been performed starting from the final configurations found in each of the TMD simulations with k = 3 and TMD time 1 ns. In the first set we have removed all the restraints from the system, in the second we have restrained C_α_ atoms. It can be seen that in half of the simulations without restraints the drug moves further towards the gate, while in the remaining ones it oscillates around the final position. Importantly, doxorubicin never goes back towards the binding pocket.
(0.07 MB TIF)

Figure S5 Drug displacement vs. subdomains movements in the periplasmic region. A) Time evolution of d_sub_, the distance between CoMs of the subdomains (shown in panel B) of AcrB mostly involved in the conformational changes during the T→O transition (see also Video S1). Larger changes in the PC1-PC2 (red line), PN2-PC2 (yellow line) and PC1-PN2 (magenta line) distances occur within the first half of the simulation, while the displacement of the substrate, d_DOX-BP_ (black dotted line, arbitrary units), essentially increases in the second half. B) Top view of the aforementioned subdomains (doxorubicin is shown as red-sticks).
(0.45 MB TIF)

Figure S6 “Peristaltic” closing of the binding pocket induces squeezing of the drug. Profile of the minimum distance d_min_ between selected pairs of residues within the binding pocket, as a function of the TMD simulation time during the T→O transition. Results are shown from eight simulations in which all heavy atoms are steered. In each panel we reported the corresponding profile of d_DOX-BP_ (red dotted lines, rescaled to fit in the graph). To better identify different behaviors, we report running averages of lenght 10 of the raw data. In 4 out of 5 the 1 ns long TMD simulations (panels A, C, D, E) a three-steps zipper-like closure of the residues lining the binding pocket can be roughly appreciated. Panel B shows a slightly different behavior, which could be viewed as a limit process in which the last step is very short. It is worthwhile to point out that also in this case the closure of the binding pocket occurs in a sequential manner, with outermost pairs F176–F615 and F136–F615 closing after the innermost ones. In panel F (5 ns long TMD) two steps can be roughly identified; again, the innermost pair F136–F628 closes before the others and the outermost 176–615 as last. The second 5 ns long simulation, panel G, shows a clearer three-steps behavior. The 10 ns simulation (panel H) also shows a zipper-like closure of residues in the binding pocket, although this appear less evident than in the previous cases. Indeed, distances between the innemost pairs, F136–F628 and F136–F617, reduce from ∼7.5 to ∼4 Å in about 3 ns, while during this time interval the outermost pairs F136–F615 and F176–F615 close only partially, going the corresponding minimum distances from ∼9 to ∼7 Å and from ∼7 to ∼5 Å respectively. Careful inspection of the graphs reveals that a further step, occurring about 1 ns later, is necessary for their complete closure. Additionally, it can be seen that the distances between outermost pairs drops at almost equivalent time. Concerning the 5 ns and 10 ns simulations, we would like to stress that, due to the large computational time needed to perform them, obtaining a relevant statistics is out of reach.
(0.14 MB TIF)

Figure S7 Effect of targeting the BP-Gate path on the movement of the drug. A) View of the BP-Gate path. Amino acids in monomers T and O are shown in blue and red, respectively (in silver the binding pocket, in transparent yellow the gate); B) Final configurations obtained respectively from TMD simulations performed with (doxorubicin colored in red) and without (cyan) bias applied to the BP-Gate path for the (T→O transition; the initial position of the drug is shown in transparent yellow). The differences in the structure of the BP-Gate path between the “standard” TMD simulation and the one without the bias on the BP-Gate path are represented using a color scale tuned on the value of the RMSD with respect to the final structure from “standard” TMD run; C) Evolution of d_DOX-BP_ (main graph) and of the RMSD (inset) as a function of TMD simulation time for the two simulations (color code as in B).
(0.69 MB TIF)

Figure S8 Effect of targeting side chains on the displacement of doxorubicin. Plot of d_DOX-BP_ as a function of the simulation time during the T→O transition for two sets of TMD simulations (averages are shown): Steering all heavy atoms (red line); Steering only C_α_ (green line).
(0.01 MB TIF)

Figure S9 Drug displacement vs. value of the force constant used for TMD. A) Plot of d_DOX-BP_ (main graph) and of the RMSD of the binding pocket (inset) as a function of the TMD simulation time during the T→O transition for two values of the force constant applied on all heavy atoms, *k* = 3 kcal mol^−1^ Å^−2^ (red full curve represents an average from all the runs having different initial velocities), *k* = 2 kcal mol^−1^ Å^−2^ (magenta dashed curve). B) Representation of the initial (yellow) and final (color code as in panel A) configurations of the drug. Note how small differences in the structure of the binding pocket affect the value of the drug displacement.
(0.02 MB TIF)

Table S1 Details of the different simulations. Shown are the cycle direction, the simulation time, the force constant, the selected atoms for the TMD, the number of simulations with the same setup, and the length of post-equilibration standard MD simulations after the targeting was finished, with or without restraints on the C_α_ atoms.
(0.08 MB RTF)

Video S1 Movement of doxorubicin and of the four subdomains PC1, PC2, PN1, PN2 extracted from one TMD simulation along the T→O transition. Firstly the whole trimer is shown, then zoom is performed on the T monomer, in the region around the drug. Doxorubicin is shown initially in blue, and becomes red following the TMD time. Some residues of the binding pocket are also highlighted.
(8.52 MB MP4)
