# Supplementary figures and images for: Functional Rotation of the Transporter AcrB: Insights into Drug Extrusion from Simulations
Source: PLoS Comput Biol. 2010 Jun 10;6(6):e1000806. doi: 10.1371/journal.pcbi.1000806 (PMC2883587; doi:10.1371/journal.pcbi.1000806)

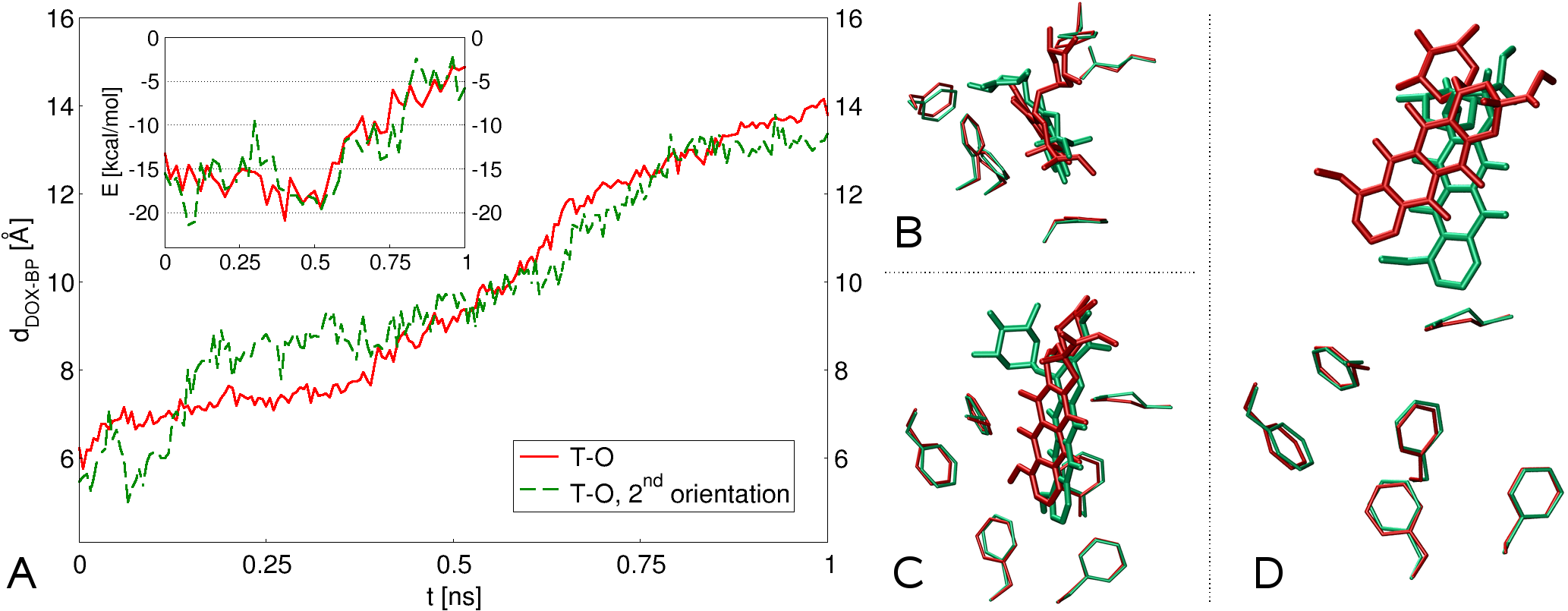

Supplement: Figure S2 — Profiles of dDOX-BP for two different conformation and orientation of the drug along the T→O transition. A) Profiles of dDOX-BP for the TMD simulation discussed in the main text (red full line) and for one simulation in which doxorubicin is in a different conformation and orientation within the binding pocket (green dashed line). In the inset is reported the behavior of the interaction energy between the drug and the residues of the binding pocket. Also shown are the two different initial positions - B) top view; D) side view - as well as (D) the final positions of the drug. (0.40 MB TIF) [file pcbi.1000806.s002.tif]

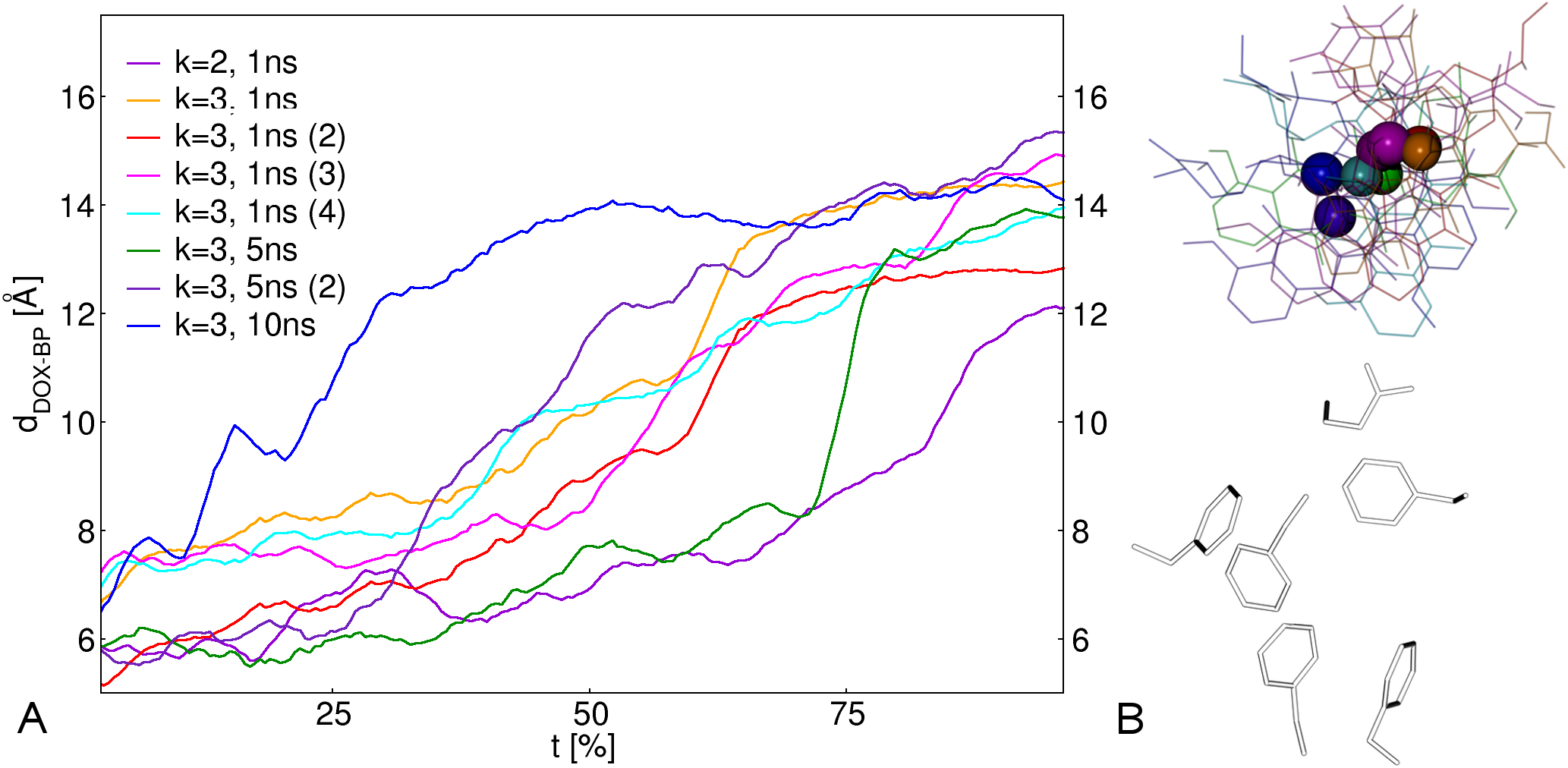

Supplement: Figure S3 — Drug displacement vs. simulation parameters along the T→O transition. A) Distance dDOX-BP as a function of the percentage of TMD simulation time for the set of simulations where all heavy atoms have been targeted. To better identify different behaviours, we report running averages of lenght 10 of the raw data. Varying the initial velocities within the set of simulations of same lenght and k value does not remarkably alter the profile of dDOX-BP. Extending the simulation time does not sensitively affect the final position of the drug, although the profile of dDOX-BP show some differences with respect to the former set; B) Final positions of doxorubicin in the same set of simulations. The CoMs of the drug are shown as filled spheres to highlight the similar displacements of the drug despite the difference which can be seen in the orientation. (0.35 MB TIF) [file pcbi.1000806.s003.tif]

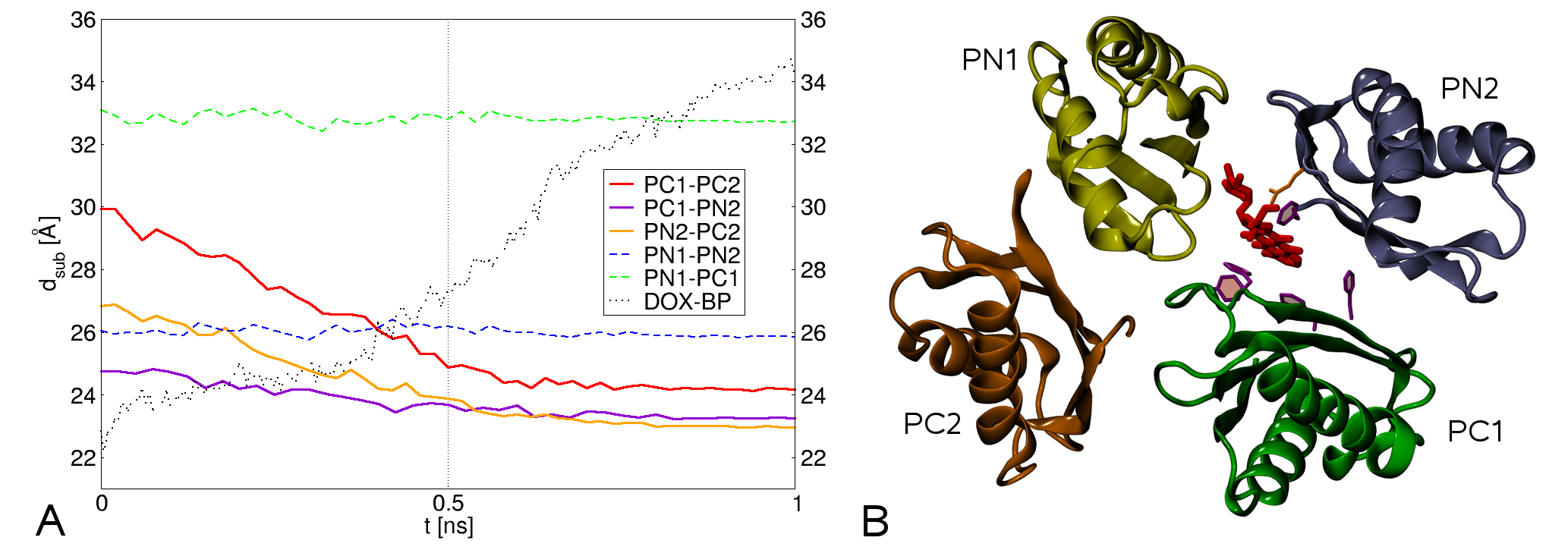

Supplement: Figure S5 — Drug displacement vs. subdomains movements in the periplasmic region. A) Time evolution of dsub, the distance between CoMs of the subdomains (shown in panel B) of AcrB mostly involved in the conformational changes during the T→O transition (see also Video S1). Larger changes in the PC1-PC2 (red line), PN2-PC2 (yellow line) and PC1-PN2 (magenta line) distances occur within the first half of the simulation, while the displacement of the substrate, dDOX-BP (black dotted line, arbitrary units), essentially increases in the second half. B) Top view of the aforementioned subdomains (doxorubicin is shown as red-sticks). (0.45 MB TIF) [file pcbi.1000806.s005.tif]

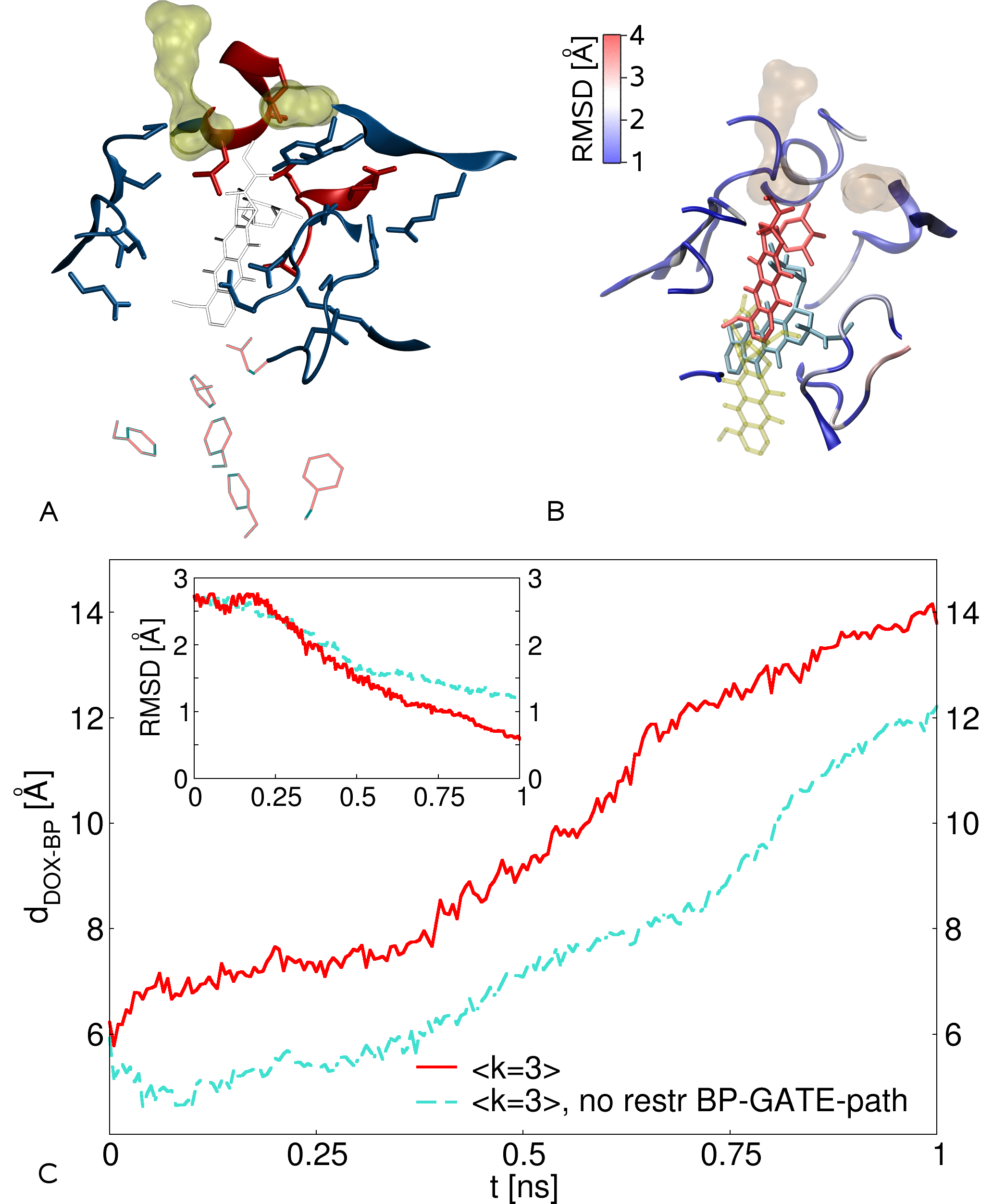

Supplement: Figure S7 — Effect of targeting the BP-Gate path on the movement of the drug. A) View of the BP-Gate path. Amino acids in monomers T and O are shown in blue and red, respectively (in silver the binding pocket, in transparent yellow the gate); B) Final configurations obtained respectively from TMD simulations performed with (doxorubicin colored in red) and without (cyan) bias applied to the BP-Gate path for the (T→O transition; the initial position of the drug is shown in transparent yellow). The differences in the structure of the BP-Gate path between the “standard” TMD simulation and the one without the bias on the BP-Gate path are represented using a color scale tuned on the value of the RMSD with respect to the final structure from “standard” TMD run; C) Evolution of dDOX-BP (main graph) and of the RMSD (inset) as a function of TMD simulation time for the two simulations (color code as in B). (0.69 MB TIF) [file pcbi.1000806.s007.tif]
