# Supplementary material for: Functional Rotation of the Transporter AcrB: Insights into Drug Extrusion from Simulations
Source: PLoS Comput Biol. 2010 Jun 10;6(6):e1000806. doi: 10.1371/journal.pcbi.1000806 (PMC2883587; doi:10.1371/journal.pcbi.1000806)
Supplement: Table S1 — Details of the different simulations. Shown are the cycle direction, the simulation time, the force constant, the selected atoms for the TMD, the number of simulations with the same setup, and the length of post-equilibration standard MD simulations after the targeting was finished, with or without restraints on the Cα atoms. (0.08 MB RTF) [file pcbi.1000806.s010.rtf]

Cycle step	TMD [ns]	katom[kcal mol-1Å-2]	Targeted atoms	Nsim	post TMD [ns]	
					Free	Restr Ca	

							
TO	1	2	heavy	1	0.25	-	
	1	3	heavy	4	2	2	
	1	3	Ca	10	5	-	
	1	3	not exit path	4	2	2	
	5	3	heavy	2	2	-	
	10	3	heavy	2	2	-	
							
TL	1	2	heavy	1	0.25	-	
	1	3	heavy	1	0.25	-	
	1	4	heavy	3	0.25	-	
LO	1	3	heavy	2	0.25	-	
	1	4	heavy	2	0.25	-	
Table S1.
